# Risk behaviors in a rural community with a known point-source exposure to chronic wasting disease

**DOI:** 10.1186/1476-069X-7-31

**Published:** 2008-06-24

**Authors:** Ralph M Garruto, Chris Reiber, Marta P Alfonso, Heidi Gastrich, Kelsey Needham, Sarah Sunderman, Sarah Walker, Jennifer Weeks, Nicholas DeRosa, Eric Faisst, John Dunn, Kenneth Fanelli, Kenneth Shilkret

**Affiliations:** 1 Laboratory of Biomedical Anthropology and Neurosciences, State University of New York at Binghamton, PO Box 6000, Binghamton, New York, 13902-6000, USA; 2 Graduate Program in Biomedical Anthropology, State University of New York at Binghamton, PO Box 6000, Binghamton, New York, 13902-6000, USA; 3 Madison County Health Department, Wampsville, New York, 13163, USA; 4 Oneida County Health Department, Utica, New York, 13501, USA

## Abstract

**Background:**

The emergence and continuing spread of Chronic Wasting Disease (CWD) in cervids has now reached 14 U.S. states, two Canadian provinces, and South Korea, producing a potential for transmission of CWD prions to humans and other animals globally. In 2005, CWD spread for the first time from the Midwest to more densely populated regions of the East Coast. As a result, a large cohort of individuals attending a wild game feast in upstate New York were exposed to a deer that was subsequently confirmed positive for CWD.

**Methods:**

Eighty-one participants who ingested or otherwise were exposed to a deer with chronic wasting disease at a local New York State sportsman's feast were recruited for this study. Participants were administered an exposure questionnaire and agreed to follow-up health evaluations longitudinally over the next six years.

**Results:**

Our results indicate two types of risks for those who attended the feast, a *Feast Risk *and a G*eneral Risk*. The larger the number of risk factors, the greater the risk to human health if CWD is transmissible to humans. Long-term surveillance of feast participants exposed to CWD is ongoing.

**Conclusion:**

The risk data from this study provide a relative scale for cumulative exposure to CWD-infected tissues and surfaces, and those in the upper tiers of cumulative risk may be most at risk if CWD is transmissible to humans.

## Background

**Figure 1:**
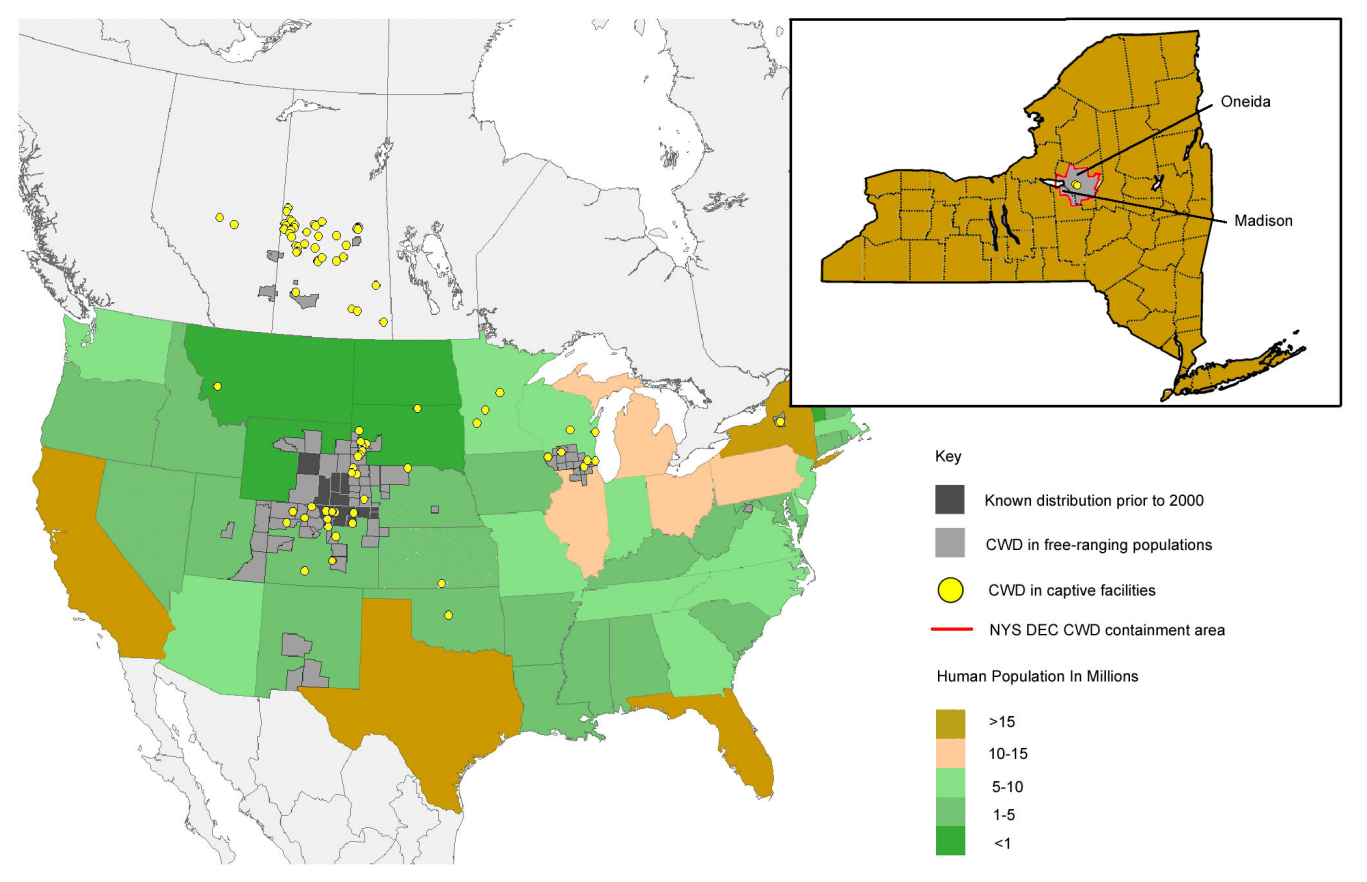
**Spatial distribution of CWD in the United States and Canada**. The CWD map is overlaid on a demographic map of the U.S. population. The insert map of New York State shows the Department of Environmental Conservation CWD Containment Area where the outbreak of CWD occurred. This depiction combines and updates information based on maps and data from the USGS National Wildlife Health Center [38], and census data from the US Census Bureau's interactive online mapping program for 2006 Population Estimates (US Census Bureau; 2006 Population Estimates; generated by Chris Reiber; using American FactFinder [39].).

Chronic Wasting Disease (CWD) was first observed in the United States in the late 1960s, though its origins are still unclear [1,2]. The term "chronic wasting disease" was first used in 1967 to describe clinical symptoms in captive Colorado mule deer [1,2]. In 1978, the disease was diagnosed as a spongiform encephalopathy [1,3]. By 1980, CWD had been described in captive elk and mule deer herds in both Colorado and Wyoming [1,2]. Subsequently in 1985, CWD was found in free-ranging elk in Wyoming, and recognized in free-ranging mule deer and whitetail deer in both states by 1990 [3]. CWD has since been described in 14 states across the US and in 2 provinces in Canada (Figure 1).

**Figure 2:**
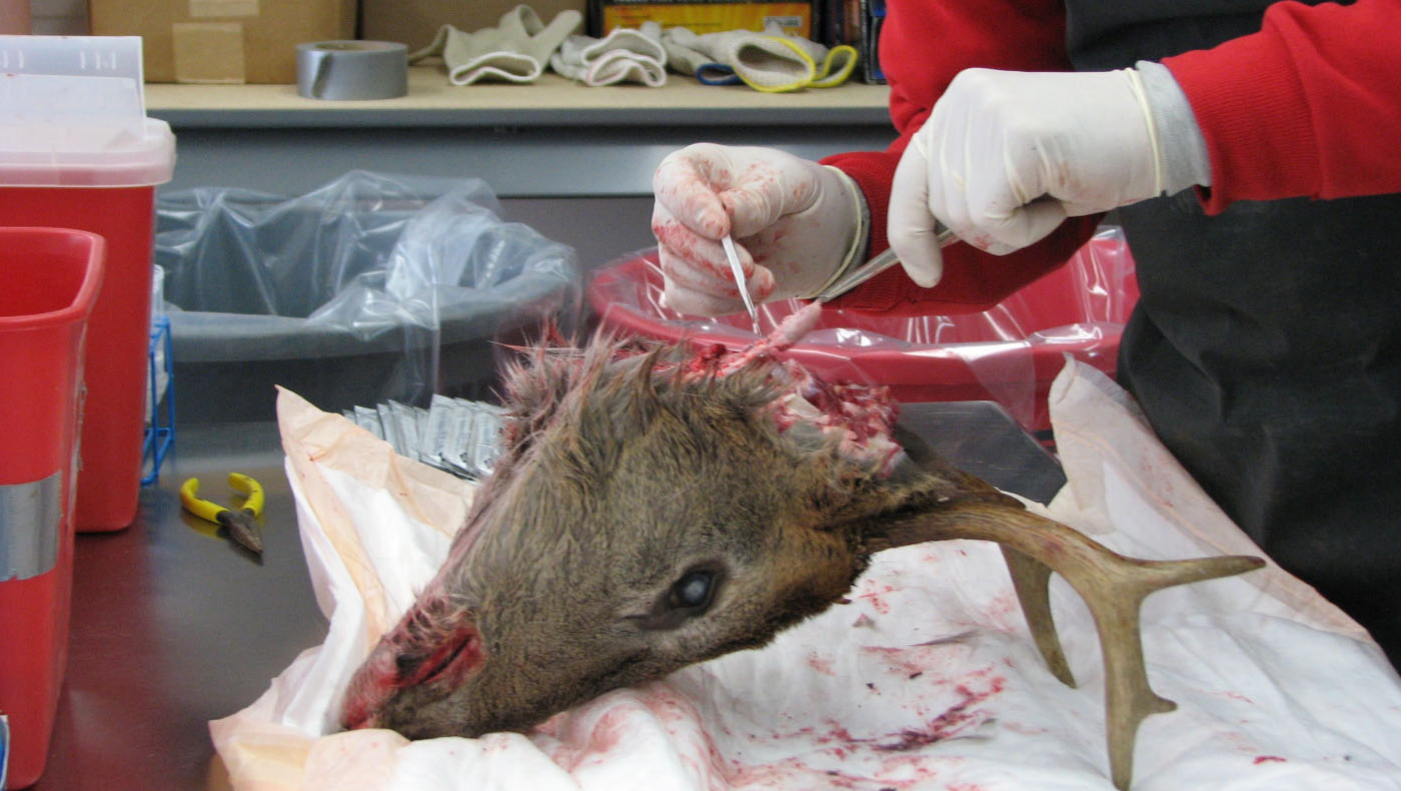
**Examination of and specimen preparation from a whitetail deer**. The check station where the deer are examined is located in the New York State Department of Environmental Conservation CWD Containment Area in Oneida County.

In April of 2002, the New York State Department of Environmental Conservation (NYSDEC) began a statewide surveillance program for CWD in whitetail deer [4]. The first positive samples (five in total) were confirmed in April 2005 from animals on two domestic deer farms in Oneida County, NY. The second deer farm, located very near to the first, received two deer that tested positive from the first deer farm. In response, the NYSDEC implemented an intensive mandatory surveillance of CWD, primarily in Oneida and Madison counties [4]. In order to monitor the wild deer herd, a CWD containment area was created encompassing approximately a 10 mile radius around the location of the CWD positive domestic deer farms in Oneida and Madison counties (Figure 1) [4]. During the initial phase of intensive monitoring (through April 30, 2005), 317 wild deer were collected and tested from this containment area as well as from the Town of Arietta in Hamilton County. Wild deer (two in total), in close proximity to the domestic deer farms, were found to be positive for CWD. During the second phase of the intensive monitoring program, all deer that died or were killed within this area were subject to mandatory testing (Figure 2). Additionally, the NYSDEC expanded its testing program statewide [4], and now the annual testing of deer for CWD includes 800–1000 animals within the containment area (Figure 1) and approximately 5000 animals statewide [4]. No additional positive deer, wild or domestic, have been found since April 2005. Details of the distribution of the deer tested across New York State for CWD can be found on the DEC website [4].

Traditions of hunting deer, elk, moose, and other cervids, and management of domestic cervid preserves bring humans into contact with animals that could have CWD. Currently, it is unclear whether CWD prions can be naturally transmitted from infected cervids to humans or to non-cervids. The question of cross-species transmission of CWD has been raised in the past [5-13]. In 2002 an unusual cluster of Creutzfeldt-Jakob Disease (CJD) – a human prion disease – developed in individuals who were avid lifelong Idaho deer hunters [14]; however, this and other reports investigating clusters of CJD have failed to establish a link with exposure to cervids through hunting or consuming the animals, or through other associated behaviors [12,14-18]. Although still unclear for CWD, epidemiological and laboratory findings have established an unequivocal link between Bovine Spongiform Encephalopathy (BSE) and vCJD [11,19-26].

On March 13, 2005, a local fire company in Oneida County, New York, hosted approximately 200–250 individuals at their annual Sportsmen's Feast, during which local wild game, including venison (deer meat), was prepared, cooked in various ways, served, and consumed by individuals primarily from Oneida and neighboring counties. Shortly thereafter, laboratory tests indicated that one of the deer served was positive for CWD [27]. Since 2002, the New York State Department of Environmental Conservation has required the mandatory testing of deer for CWD harvested from a domestic deer farm. However, there is no requirement that the meat not be consumed before the results are made available and thus reaction to the finding that the deer tested positive ranged from unconcerned to anger. Feast attendees were exposed to the contaminated meat through a variety of activities, including butchering and processing, cooking, consumption of venison, and/or through contact with contaminated surfaces. This incident represents the only known large scale point-source exposure of humans to an animal with confirmed CWD.

In response to this known point-source exposure, the Oneida County Health Department and the State University of New York at Binghamton (SUNY) proposed and launched the Oneida County Chronic Wasting Disease Surveillance Project, a cohort study designed to examine a natural experimental model of human exposure to CWD. The objective of the study are to determine the potential human health risks associated with exposure to CWD-contaminated cervids by following the events and health outcomes of attendees at the Sportsmen's Feast. Study participants are being followed for a minimum period of six years from the time of exposure. This is based on the minimum incubation period and earliest age at onset for known human prion diseases including vCJD [11] and kuru [28-31]. The current report describes the initial results of a risk analysis based on behaviors associated with the wild game feast in upstate New York.

## Methods

The exposed cohort, whose names are held confidentially by the Oneida County Health Department, were contacted by mail about an informational meeting held in Oneida County in November, 2005. The project was approved by the Institutional Review Board at SUNY Binghamton. Questionnaires and informational packets were distributed either by mail or in person to those who expressed an interest in participating in the surveillance project. From January to June 2006, SUNY Binghamton research assistants conducted additional follow-up mailings and telephone calls to those who indicated an interest in participating. The information from the project's 81 recruited participants was entered into an Excel database, using standard double-entry, cross-check and proof-reading techniques to ensure accuracy. Statistical Package for the Social Sciences (SPSS) version 13.0 was used to analyze the data from the participant questionnaires.

The epidemiological questionnaire consisted of 54 questions, divided into four sections: demographic information, participation in feast activities, participation in general activities (including hunting and occupations), and current health information. The questionnaire was designed to obtain information to allow risk assessment at two levels: participation in feast activities termed *Feast Risk*, and other activities outside of the feast, termed *General Risk*.

Eleven variables were chosen as potential risk factors in the analysis of *Feast Risk*. These variables were selected based on known transmission routes from studies of other transmissible spongiform encephalopathies [11,19-26,28,32,33]. *Feast Risk *included eleven different activities that took place in preparation of, or at the Sportsmen's Feast. They include: venison consumption, deer butchering, sustaining wounds while butchering, not wearing gloves while butchering, handling raw meat, eating raw meat, eating chops, eating tongue, cooking, not wearing gloves while cooking, and sustaining injuries while cooking.

In addition to the risk factors at the feast, seven behaviors were considered risk factors for analyzing *General Risk*. These included: hunting deer, hunting in the containment area, eating venison "often" (by subjective self-report), butchering deer, field dressing deer, removing antlers, and not wearing gloves while butchering.

## Results

### General Characteristics of Study Participants

The feast participant sample is composed of 86.4% males and 13.6% females (N = 81). Based on self-report, the majority of the participants identified themselves as white (89.8%), with 1.3% identifying as Native Hawaiian, and 1.3% as Native American. Most participants attended the Feast (96.3%) and of those attending most were accompanied by friends or family (78.9%). A total of nine different counties of residence in New York and two outside the state were reported, but the majority live in two New York counties: 53 in Oneida County (65%), and 17 in Madison County (21%). Ages ranged from 10 to 82 years, with a mean of 48 years.

A total of 13.6% of participants reported being diabetic (Type II). Only 2.5% had been diagnosed previously with a neurological disease, but 19.8% reported a family history of neurological disease, with Alzheimer's disease being the most common condition (13.6%).

### Feast Risk

**Figure 3:**
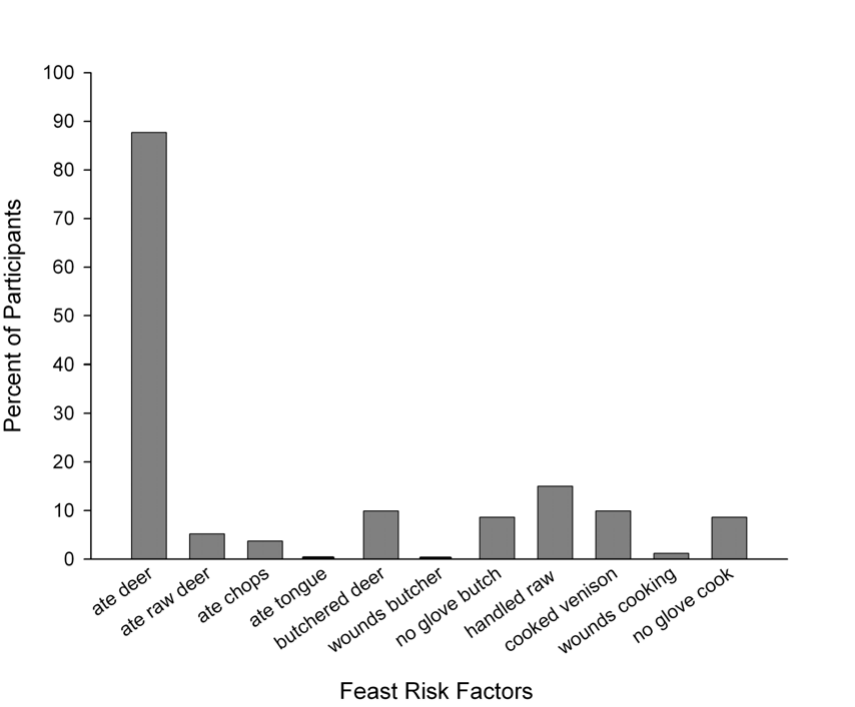
Percent of participants (N = 81) affected by each Feast Risk factor.

**Figure 4:**
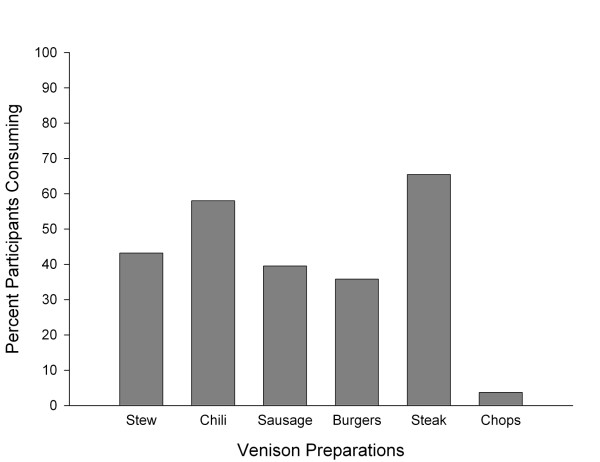
Percent of participants (N = 81) consuming various types of venison preparations at the sportsman's feast.

Figure 3 shows the percentage of the 81 participants who sustained each individual risk factor at the Feast, with 87.7% of the attendees eating venison. Table 1 shows that most of the 81 participants had only one *Feast Risk *factor (69.1%), most commonly eating venison, while 9.9% had none. Participants who reported venison consumption reported that they ate it prepared in different ways, most commonly as chili and steak (Figure 4). The only organ meat consumed was deer liver (1.3%). No other organ meat such as tongue, heart or kidney was reported to have been consumed.

**Table 1:** Number of risk factors for study participants (N = 81).

Risk category	# risk factors	n	cum n	%	cum %
Feast Risk	0	8	8	9.9	9.9
	1	56	64	69.1	79
	2	6	70	7.4	86.4
	3	4	74	4.9	91.4
	4	1	75	1.2	92.6
	5	0	75	0	92.6
	6	4	79	4.9	97.6
	7	2	81	2.5	100
General Risk	0	19	19	23.5	23.5
	1	6	25	7.4	30.9
	2	2	27	2.5	33.4
	3	7	34	8.6	42
	4	9	43	11.1	53.1
	5	15	58	18.5	71.6
	6	13	71	16	87.7
	7	10	81	12.3	100

Seventeen individuals (21%) had multiple *Feast Risk *factors, having both eaten venison and participated in some other way(s) in the butchering and/or preparation of the venison. Among participants who attended the feast, only one (1.3%) participated in field dressing deer served at the feast, and no one recalled field dressing the contaminated animal. Individuals involved in the preparation and processing of the deer reported they: butchered (n = 8; 9.9%), handled raw meat (n = 12; 15%), and cooked venison (n = 8; 9.9%). No one reported sustaining a cut or any other injury while butchering, but only one of the eight who butchered wore gloves. However, two individuals stated that they had cuts on their hands at the time of the feast (2.5%), while 32.5% do not recall whether they did. Of the eight participants involved in cooking venison, one reported sustaining an injury while cooking, and one also indicated that they wore gloves while cooking.

### General Risk

**Figure 5:**
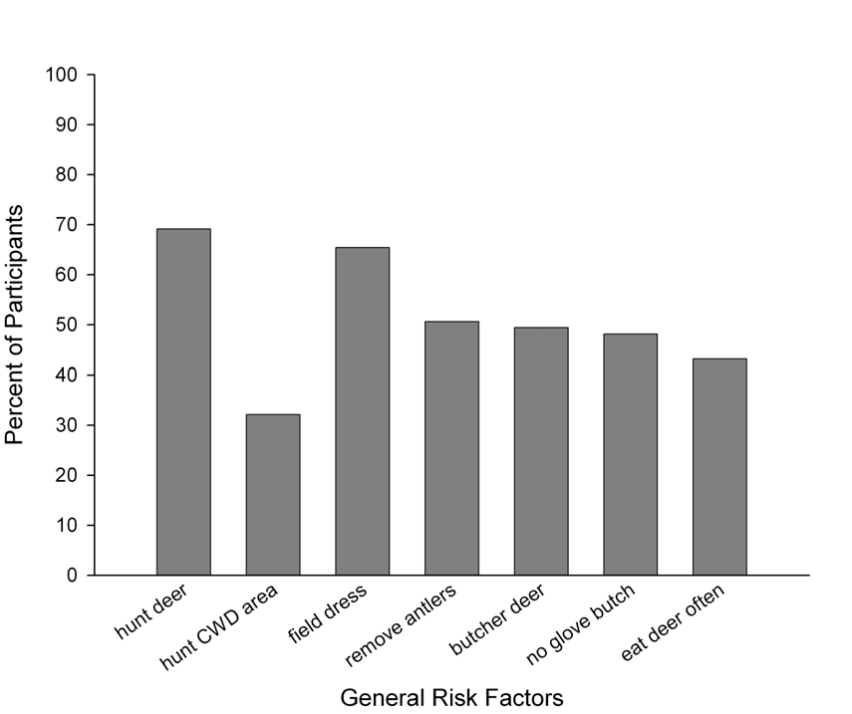
Percent of participants (N = 81) affected by each General Risk factor.

Results regarding *General Risk *factors are presented in Figure 5. In relation to deer hunting practices, of the 81 participants, 69.1% hunted deer, and of those who hunted, 80.4% harvested a deer between the years 2000–2005. Of those who hunted, 25.0% reported hunting *only *in Oneida County. Furthermore, a total of 32.1% of the participants indicated that they hunted in the Oneida County CWD containment area, although not exclusively. Of those who hunted, 96.4% field dressed, 75.5% removed antlers and 70.9% butchered harvested deer. However, only 26.4% of them wore gloves while butchering deer. Of those who hunted, 92.7% consumed the deer they killed, and 96.3% of all feast participants reported eating venison outside of the Sportsmen's Feast. Of the 81 participants, 24.6% also reported eating venison from other states. In addition to hunting, the 81 participants reported general contact with animals, including deer (39.5%), cattle (13.6%), sheep (1.2%), alpacas (1.2%), and other animals (9.9%).

## Discussion

The purpose of this report is to describe the events surrounding the exposure of a large cohort of individuals to a CWD-infected animal, and present information on risk behaviors for activities at the feast and general hunting behaviors outside the feast. This study focuses on two levels of risk, that specific to activities at the feast, *Feast Risk*, and activities outside the feast, *General Risk*. Because CWD has not been definitively linked to the development of a prion disease in humans, the risk behaviors evaluated reflect those associated with known prion disease transmission routes [11,19-26,28-33].

Studies have shown that CWD can be experimentally transmitted to voles [13], non-human primates [7], cattle [5,8], and sheep [9]. Since transmissible spongiform encephalopathies have the ability to cross species barriers and are resistant to degradation [5-10,13,16,18,28,29,32-34], food chain transmission of prion diseases is a growing human and animal health concern. Infected prions are found in the blood, skeletal muscle, and saliva as well as the central nervous system of infected animals [5,10,33,35]. Contact with these tissues is a likely mode of transmission of CWD from animal to animal, and could potentially present a risk to humans. It is currently considered unlikely that CWD can cross the species barrier to humans [12,14,18]. However, if it can, those with multiple risk factors may be most vulnerable. The information presented above provides a relative scale for cumulative exposures to CWD-infected tissues and surfaces.

Since CWD has spread from regions of the Midwest with generally low human population densities to high-density regions of the Eastern U.S. (New York and West Virginia), direct contact between infected cervids and humans has increased. Additionally, the potential for cross-species transmission of CWD from deer to cattle to humans may be increased as a result of increasing contact between large herds of potentially infected cervids and cattle in pastures on smaller Eastern U.S. farms.

## Conclusion

The Oneida County CWD Surveillance Project introduced here, and the data reported in this paper, provides the first step in developing a natural experimental model of possible transmission of CWD prions from deer to humans [36,37] with a known point-source exposure. Surveillance of the cohort will continue for a minimum of six years (and likely extended) through annual follow-up questionnaires including self-report health information. Since human prion diseases are reportable in New York State, the Oneida County Health Department and health departments in other neighboring counties will be especially vigilant in this surveillance effort. This prospective cohort study will provide new information previously unavailable from retrospective studies where the CWD status of cervids was unknown and hunting behaviors only evaluated retrospectively many years after onset of cases of CJD.

## Abbreviations

CJD: Creutzfeldt-Jacob Disease; CWD: Chronic Wasting Disease; NYSDEC: New York State Department of Environmental Conservation; SUNY: State University of New York; vCJD: Variant Creutzfeldt-Jacob Disease

## Competing interests

The authors declare that they have no competing interests.

## Authors' contributions

RMG conceived, designed and coordinated the study and prepared the manuscript, CR coordinated the data analysis, analyzed the data and prepared the manuscript, JW coordinated the field research, and HG and SS followed-up with the participants and conducted the analysis, MA, KN, SS, and SW conducted follow-up of the participants, data analysis and preparation of the manuscript, ND, EF, JD, KF and KS coordinated the town hall meeting, distribution of recruitment letters, development and dissemination of the questionnaires to the participants, and preparation of the manuscript. All authors read and approved the final manuscript.
